# Congratulations to the new editor

**DOI:** 10.4103/0972-124X.44084

**Published:** 2008

Its my pleasure to reach out to the fellow members of our esteemed society through this journal. I take this opportunity to congratulate our new editor Dr. Arunachalam on bringing out this issue and bringing ISP journal back on track.

The journal is truly a window to the activities of the society and reflects the academic and research standards in our country.Periodontology and Implantology is in its most exciting era with tremendous advances in research, biomaterials and regenerative therapies. I sincerely request all the members to contribute by sending their valuable suggestions and articles.

We have already started the years work with the post graduate workshop at Udaipur in July followed by oral hygiene day celebrations, as well as a scientific programme to be implemented covering the multiple venues across the country and to crown it all the national conference scheduled in November at Chandigarh.

I look forward to your encouragement continuous support and constructive criticism in all our endeavors.

With good wishes.

Professionally yours,

Praveen B. Kudva
Acting President,
Journal of Indian Society of Periodontology
E-mail: smilesindia@gmail.com

